# Effect of the bacterium *Serratia marcescens* SCBI on the longevity and reproduction of the nematode *Caenorhabditis briggsae* KT0001

**DOI:** 10.1186/1756-0500-5-688

**Published:** 2012-12-20

**Authors:** Jeremiah D Lancaster, Budour Mohammad, Eyualem Abebe

**Affiliations:** 1 Department of Biology and Marine Environmental Science, Elizabeth City State University, Weeksville Road, Elizabeth City, NC 27909, USA

**Keywords:** *Caenorhabditis briggsae*, *Serratia*, Symbiosis, Fitness, Nematode-bacterial associations

## Abstract

**Background:**

Extensive research effort has advanced our understanding of *Caenorhabditis* as a model system, but its natural association with bacteria remains to be explored in an ecological context. Explored associations vary vastly from mutualistic to parasitic. *Serratia marcescens* has been shown to be pathogenic to *Caenorhabditis* with a fitness cost*.* The recent isolation of an entomopathogenic *Caenorhabditis briggsae* KT0001*/S. marcescens* SCBI association from the wild has allowed us to examine under laboratory conditions whether such an association poses a serious cost to *Caenorhabditis* as previously surmised for other *Serratia*.

**Results:**

A fecundity table of *Caenorhabditis briggsae* KT0001 fed on *S. marcescens* SCBI and the control fed on *E. coli* OP50 is presented. We found no significant difference in survivorship or total fecundity between the *S. marcescens* SCBI fed and *E. coli* OP50 fed *Caenorhabditis briggsae* KT0001. Only the mean onset of reproduction was significantly different between the two groups with *E. coli* fed *C. briggsae* maturing earlier (2.12 days) than those fed on *Serratia* (2.42 days)*.*

**Conclusion:**

*S. marcescens SCBI* is not highly pathogenic to *C. briggsae* KT0001 indicating that the entomopathogenicity reported for this association may be beneficial for both the nematode and bacteria. In light of the fact that hitherto conducted experimental tests conform to widely held view that *Serratia* are highly pathogenic to *Caenorhabditis*, the absence of a high fitness cost for *C. briggsae* we report here may indicate that this entomopathogenic association is non-transient suggesting nematode/bacterial associations in the wild may vary greatly. Consequently, broad generalizations about nematode/bacterial associations should be interpreted with care.

## Background

Nematode-bacterial associations have proven to be complex but our knowledge of their natural occurrence is limited. Two well-studied cases of nematode-bacterial associations are those of entomopathogenic nematodes and the case of *Wolbachia* and filarial worms [1]. Nematodes in these associations are considered obligate animal parasites. These studies demonstrated that bacterial associations could give the nematodes an advantage in ensuring the availability of food and the survival of the nematode species, and could even impact developmental processes of the host. Despite extensive studies on these well-characterized nematode-bacterial associations, there is very little data on bacteria associated with free-living nematodes. Despite extensive research, our understanding of the ecology of *Caenorhabditis* in its natural settings is little explored [2]. For example, a recent study has changed the long-standing view that *Caenorhabditis* are inhabitants of organic rich soil environment to one where they are considered “fruit-worms” [3].

Bacterial associations of *Caenorhabditis* can vary vastly: they may be mutualistic, parasitic, or may be commensal where worms serve as vectors of pathogenic bacteria as in the case of *C. elegans* and *Salmonella*[4]. *Pseudomonas* strain DSS73 has been shown to associate mutually with *C. elegans*, increasing their survival in complex multispecies environments [5].

Although *Caenorhabditis* has long been considered to maintain necromenic associations with invertebrates [6], a recent report demonstrated, in laboratory experimental setting, a more active killing ability than the usual necromeny where an *C. briggsae*/*Serratia marcescens* association was shown to kill Galleria [7].

*Serratia marcescens* is a cosmopolitan bacterium widely isolated from soil, water, plants and insects, and exhibits pathogenic or saprophytic characteristics [8]. Its opportunistic pathogenicity in humans is also well recognized. Three recent studies have reported the association of *Serratia* with nematodes [7,9,10]. Those nematodes were isolated using standard *Galleria* traps and in all the three cases the association afforded the ability to kill the insect in laboratory setting which was mutually beneficial to the nematodes and bacteria. These laboratory associations are tripartite and complex involving the nematode that goes into the host insect, and symbiotically associated bacteria that kills the insect and in the process serves as food and creates a favorable environment for the nematodes to reproduce and complete their life cycle. Symbiosis, in biological context, is a broad term that represents long term associations between different biological species and could be one of the three main types: mutualism, commensalism, and parasitism.

Pathogenic bacteria are related to increased mortality, and reduced fecundity [11-13]. On the other hand, within the specific association of *Caenorhabditis* with *Serratia marcescens*, experimental tests of pathogenicity of the bacteria towards *Caenorhabditis* were shown to be strain-specific. *Serratia marcescens* Db10, for example, is known to kill *Caenorhabditis*[11,13], but *S. marcescens* SCBI did not kill the worms, instead it afforded them insect killing ability [7]. Given that *Serratia* is pathogenic to *Caenorhabditis* in experimental settings, investigating the cost of this relationship is relevant to a better understanding of the association and its historical progress. Also, it has been shown that the progeny count of *C. elegans* improved by 58% when fed live bacteria rather than feeding on dead bacteria [14] indicating that food quality impacts fitness in *Caenorhabditis*. As fitness is a key ecological parameter in measuring the success of any organism, and can be measured using reproductive rate, the objective of this research was 1) to evaluate if feeding on *S. marcescens* SCBI has a fitness cost for *C. briggsae* KT0001 and, 2) to estimate life history characteristics of the tropical strain – *C. briggsae* KT0001 – under laboratory conditions.

## Results

### Survivorship

**Figure 1:**
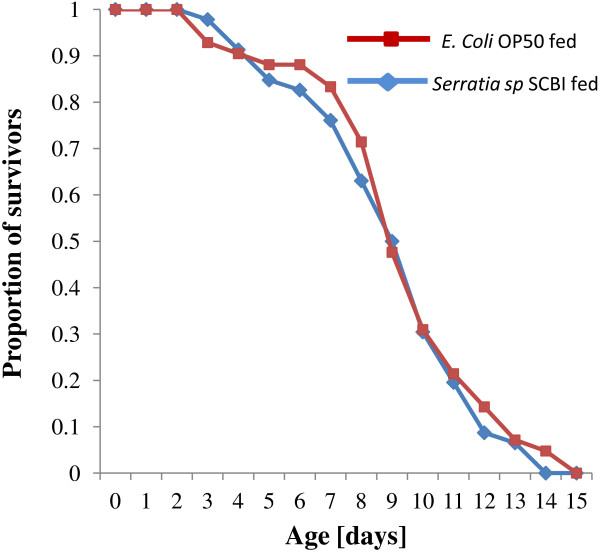
**Survivorship curve of *C. briggsae *fed on either *S. marcescens *or *E. coli *OP50. **Curves represent the percentage of survivors as a function of time. Sample sizes were n = 46 (*E. coli *OP50) and n = 42 (*S. marcescens*).

Juvenile mortality was not observed in either the *E. coli* OP50 or *S. marcescens* SCBI fed *C. briggsae*, with all 88 juveniles surviving to maturity. Most of the reproductive output was complete by day 5 for both groups, and the survival rate for each group at that time was 85% for the *E. coli* OP50 fed population and 88% for the *S. marcescens* SCBI fed population. Both *S. marcescens* SCBI fed and *E. coli* OP50 fed populations of *C. briggsae* exhibited Type I survivorship curve (Figure 1) typical for the species, with most surviving until near end of life span. We found no significant difference in survivorship between the two groups of *C. briggsae* (log rank test, Chi sq = 0.4, p = 0.543): median survival rate (TL_50_) was 9 days. Average lifespan was 9.1 ± 2.8 days with a maximum lifespan of 14 days for the *E. coli* OP50 group compared to an average lifespan of 9.4 ± 2.9 days and maximum lifespan of 15 days in the *S. marcescens* SCBI group.

### Reproduction

**Figure 2:**
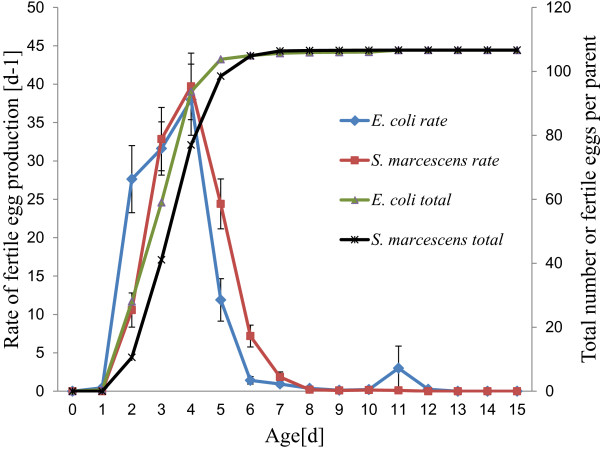
**Fecundity of *C. briggsae *fed on either *E. coli *OP50 *or S. marcescens *SCBI. **Sample sizes were n = 46 (*E. coli *OP50) and n = 42 (*S. marcescens *SCBI).

Reproduction was observed over a period of 15 days (day 0–14) with 9,385 offspring counted for all 88 individuals. Onset of reproduction was significantly different (p < 0.05) with mean onset of reproduction at 2.12 days for the *E. coli* OP50 fed population and at 2.42 days for the *S. marcescens* SCBI fed population. A comparison of fecundity of *C. briggsae* fed on *E. coli* OP50 and fed on *S. marcescens* SCBI is shown in Figure 2. Fecundity was most pronounced between days 2 and 5, with day 4 marking the peak for both groups. Maximum age-specific fecundity peaked at a mean of 38 offspring/survivor for *E. coli* OP50 fed population and 40 offspring/survivor for *S. marcescens* SCBI fed population (Table 1). This difference was not statistically significant (*p* = 0.222).

**Table 1:** **Life table and fecundity schedule of *C. briggsae *fed on *E. coli *OP50 or *S. marcescens *SCBI**

	***E. coli *OP50**					***S. marcescens *SCBI**				
**X**	**n**_**x**_	**l**_**x**_	**m**_**x**_	**l**_**x**_**m**_**x**_	**xl**_**x**_**m**_**x**_	**n**_**x**_	**l**_**x**_	**m**_**x**_	**l**_**x**_**m**_**x**_	**xl**_**x**_**m**_**x**_
0.00	46.00	1.00	0.00	0.00	0.00	42.00	1.00	0.00	0.00	0.00
1.00	46.00	1.00	0.46	0.46	0.46	42.00	1.00	0.00	0.00	0.00
2.00	46.00	1.00	27.63	27.63	55.26	42.00	1.00	10.57	10.57	21.14
3.00	45.00	0.98	31.62	30.94	92.81	39.00	0.93	32.85	30.50	91.50
4.00	42.00	0.91	37.98	34.67	138.69	38.00	0.90	39.71	35.93	143.72
5.00	39.00	0.85	11.90	10.09	50.43	37.00	0.88	24.41	21.50	107.50
6.00	38.00	0.83	1.42	1.17	7.04	37.00	0.88	7.19	6.33	38.00
7.00	35.00	0.76	0.94	0.72	5.02	35.00	0.83	1.86	1.55	10.83
8.00	29.00	0.63	0.38	0.24	1.91	30.00	0.71	0.20	0.14	1.14
9.00	23.00	0.50	0.13	0.07	0.59	20.00	0.48	0.10	0.05	0.43
10.00	14.00	0.30	0.21	0.07	0.65	13.00	0.31	0.15	0.05	0.48
11.00	9.00	0.20	3.00	0.59	6.46	9.00	0.21	0.11	0.02	0.26
12.00	4.00	0.09	0.25	0.02	0.26	6.00	0.14	0.00	0.00	0.00
13.00	3.00	0.07	0.00	0.00	0.00	3.00	0.07	0.00	0.00	0.00
14.00	0.00	0.00	0.00	0.00	0.00	2.00	0.05	0.00	0.00	0.00
15.00	0.00	0.00	0.00	0.00	0.00	0.00	0.00	0.00	0.00	0.00
∑			115.92	106.65	359.58			117.14	106.65	415.01

The fixed time interval, x = age in days; n_x_ = number of surviving individuals; l_x_ = proportion of survivors remaining; m_x_ = age-specific fecundity; ∑m_x_ = total fertility rate (TFR); ∑l_x_m_x_ = net reproductive rate (R_0_).

We plotted total number of produced eggs against total lifetime (Figure 2) as suggested by Davies and Hart [14] and Muschiol et al. [15], to explore whether the two estimates are related. Spearman’s rho was weak but significant in both groups (*E. coli* OP50: r_s_ = 0.45, p < 0.01; *S. marcescens* SCBI: r_s_ = 0.43, p < 0.01).

The fecundity schedule of *C. briggsae* KT0001 is given in Table 1. The sum of age-specific fecundity produced total fertility rates of 116 for the *E. coli* OP50 fed group and 117 for the *S. marcescens* SCBI fed group with net reproductive rates (R_0_) of 107 for both groups (Table 1). The minute discrepancies are due to the minutia difference in age-specific mortality.

Cohort generation time (T_C_) and intrinsic rate of natural increase (r_m_) differed slightly between the two groups with the *E. coli* OP50 fed *C. briggsae* KT0001 having a cohort generation time (T_C_) of 3.372 days and an intrinsic rate of natural increase (r_m_) of 1.385; and the *S. marcescens* SCBI fed *C. briggsae* having a cohort generation time (T_C_) of 3.892 days and an intrinsic rate of natural increase (r_m_) of 1.200. Population doubling times for *C. briggsae* KT0001 were calculated to be 12.0 hours for the *E. coli* OP50 fed group and 13.9 hours for the *S. marcescens* SCBI group. This is in comparison to known *C. elegans* populations doubling times [15] of 11.4 hrs (N2) and 12.1 hrs (MY6) when fed *E. coli* OP50 at 20 ± 1°C.

## Discussion

The relationship between *Caenorhabditis* and *S. marcescens* has been shown to be strain and genotype-specific in lab experimental studies. For example, *S. marcescens* Sma13 is more virulent than *S. marcescens* ATCC274 to *C. elegans* MY10, but these results are contrary to what is reported for *C. elegans* MY20. *C. elegans* MY15 is also unique, with higher susceptibility to *S. marcescens* ATCC274 than to *S. marcescens* Db11, since most other *C. elegans* strains are more susceptible to Db11. It is not known if such strain- and genotype-specific relationships exist under natural conditions [16]*.* However, the isolation of *S. marcescens* SCBI from *C. briggsae* KT0001 [7] in the framework of exploring nematode-bacterial associations, enabled us conduct our current study and the results give strong support to co- evolutionary interactions as was suggested by Schulenburg and Ewbank [16].

### Survivorship

Survivorship is known to be markedly reduced for *Caenorhabditis* fed on pathogenic bacteria when compared to those fed on *E. coli* OP50 control [11,12,16-23]. This has been repeatedly shown on various strains of *C. elegans* with *S. marcescens*[11,16,18-20]. Sifri et al. [20] surveyed various survival rates for *C. elegans* fed on *S. marcescens* and found median survival rates (TL_50_) of 2–6 days; similar results were observed (TL_50_ = 4–5 d) by Pradel et al. [19] using two different strains of *S. marcescens*. These results differ greatly from our current results with our TL_50_ of 9 days for both groups of *C. briggsae* KT0001. The starkly similar survivorship curves of our *C. briggsae* grown on *S. marcescens* SCBI and *E. coli* OP50 may provide a strong indication that nematode-bacterial association in general and *Caenorhabditis*/*S. marcescens* relationships in particular, in the wild, may differ greatly from our current understanding based solely on laboratory experimental interactions.

### Reproduction

Fecundity has been shown to be species specific in *Caenorhabditis* in the context of interactions with pathogenic bacteria. Rae et al. [23] showed a significant reduction in fecundity associated with high mortality before and during peak reproduction, while Baeriswyl et al. [12] showed no significant difference in fecundity in relation to various strains of *E. coli*, had an earlier onset of reproduction and less mortality prior to peak reproduction. This suggests that fecundity is reduced in *Caenorhabditis* by highly pathogenic bacteria due to age-specific morbidity and mortality. Our results showed no significant difference between net reproductive rate or total fertility which, in light of Rae et al. [23], precludes the *C. briggsae* KT0001/*S. marcescens* SCBI relationship from the league of the highly pathogenic relationships reported elsewhere. Differences in mean onset of reproduction were significant (2.12 days for the *E. coli* OP50 fed group and 2.42 days for the *S. marcescens* SCBI fed group). This shift reduced overall fitness by increasing cohort generation time (T_C_) and reducing intrinsic rate of natural increase (r_m_). This shows that although the relationship shows a marked difference from entirely pathogenic forms, it is not entirely mutualistic either at a level comparable to what we observe in archetypical entomopathogenic nematodes and their symbionts. Life history theory suggests this shift may be due to an investment in defense mechanisms [24]. The maintenance of net reproductive rate and total fertility, despite investments in defense mechanisms, supports long term co-evolutionary interactions consistent with a non-transient relationship.

Symbiotic relationships are often complex and range from parasitic to mutualistic. The fitness benefits of microbial symbionts to the symbiont partner is recently shown to be context dependent [25] where environmental factors may affect rapid shifts between mutualism and parasitism [26]. Weeks et al. [27] showed that such evolutionary shifts in nature can be achieved within a relatively short period of time. Moreover, the theoretical divide between parasitism and mutualism has blurred over the last decade in that mutualists are selected to minimize their costly contributions while maximizing their benefits from the relationship – a processes resembling the ‘antagonistic arms race’ model except here the symbiont pair are selected to exploit each other while minimizing loss on their side [28]. Sachs et al. [28] argues that “transitions to host association might be constrained only by access to and compatibility with horizontally transferred loci that engender host-association traits”. Previous reports of some *Serratia marcescens* as pathogenic to *Caenorhabditis* species, and recent findings that many species of *Serratia* are symbiotically associated with invertebrate hosts and our findings of a *Serratia marcescens* SCBI associated with *C. briggsae* KT0001 that experimentally was able to kill Galleria are in support of an unexplored diversity of *Serratia* within an umbrella species name – *Serratia marcescens*. In addition, Sachs’ et al. [28] predictions coupled with the fact that nematodes and bacteria are two species-rich and ancient groups [29,30] imply that expecting to find a diverse array of hitherto undiscovered nematode-bacterial associations is not unreasonable.

## Conclusion

*S. marcescens* SCBI does not affect the longevity of *C. briggsae* KT0001. As a result, the association between the two is likely to impose a minimal reproductive cost to the nematode, an indication that although such associations between *Caenorhabditis* and *Serratia* have been widely reported to affect the fitness of the nematode through bacterial pathogenicity, our findings show the *C. briggsae* KT0001/*S. marcescens* SCBI association to be an exception to the rule.

## Methods

### Culture maintenance

We used *C. briggsae* KT0001 from the original collection at the Hubbard Center for Genome Studies, University of New Hampshire. *C. briggsae* were cultivated from frozen stocks, and were maintained on Nematode Growth Media (NGM) by transferring a number of adults every week onto a NGM plate with fresh food [31,32]. The primarily goal of the experiment was to test the implications of the two types of bacterial food sources, *S. marcescens* SCBI and *E. coli* OP50, on nematode reproductive potential. As a result, nematodes were maintained on two sets of cultures: one seeded with 20 μL *E. coli* OP50 starting from the initial thawing to the completion of the experiment and the second on *S. marcescens* SCBI.

Nematode Growth Gelrite (NGG) media was prepared following the protocol described by Muschiol & Traunspurger [33]. NGG was made by replacing agar with 1.5 g of gellan gum in NGM. The viscosity of this media allowed individuals to move freely throughout the media whilst preventing bacteria from settling. Its transparency also allowed easy transfer of individuals and counting of juveniles.

### Food preparation

*E. coli* OP50 and *S. marcescens* SCBI were inoculated into sterile LB broth and put on a shaker for 24 hours at 20°C to allow for cell growth. After 24 hrs, bacteria were concentrated to 10^10^cells/mL using an absorption (OD_600_) vs. bacterial concentration curve [33].

### Experimental setup

Our experimental setup closely followed that described by Muschiol and Traunspurger [15]. Sets of 24 well multi-well plates were used with 10 μL mixture drops (i.e. 8 μL of NGG with 2 μL of bacterial food) added to the center of the lid of each well of the multi-well plate. The wells consisted of a 1 mL mix of cellulose and sterile water in order to maintain humidity and prevent the drops from drying out. A synchronous juvenile worm was added to each drop. Every 24 hours we transferred each worm to a new NGG drop until all died. All NGG drops were kept at 20°C in the environmental chamber for an additional 24 hours after the individual was transferred or died. This 24 hr time allowed eggs to hatch. We counted only juveniles as an indicator of fertile eggs. Twenty-four hours after transfer, juveniles were stained with 10 μL of rose Bengal, squashed with a 30 mm round cover slip, and counted under a 40X magnification dissecting microscope (Olympus SZX10 Stereomicroscope).

### Data processing

Life tables and fecundity schedules (Table 1) were constructed to find cohort generation time (T_C_), intrinsic rate of natural increase (r_m_), and population doubling time (PDT) using the following equations:

(1) $$\text{TFR}=\sum m_{\times }$$

(2) $$R_{0}=\sum_{\times }m_{\times }$$

(3) $$T_{c}=\sum {x\;}_{x}m_{x}/R_{0}$$

(4) $$r_{m}=\left({\ln\;R}_{0}\right)/T_{c}$$

(5) $$\mathrm{PDT}=\left(\ln\;2\right)/r_{m}$$

x = time [d]

l_x_ = age specific survival probability

m_x_ = age specific fecundity

TFR - total fertility rate

R_0_ = net reproductive rate

T_C_ = cohort generation time

r_m_ = approximate intrinsic rate of natural increase

PDT = population doubling time

The net reproductive rate (R_0_) is the average number of offspring from an individual in a lifetime, and dependent on age specific mortality. Cohort generation time (T_C_) is the mean age of a female cohort at reproduction. The intrinsic rate of natural increase (r_m_) is a measure of growth rate and fitness in a population of stable age distribution, and unlimited growth [15].

Statistical analysis was carried out using Microsoft Excel (2007) or manually. Unpaired data was compared using student’s *T*-test. Survivorship was compared using a log-rank test, and total number of offspring vs. total lifetime was correlated using Spearman’s rho.

## Competing interests

The authors declare that they have no competing interests.

## Authors’ contributions

JDL and BM carried out the experiment and drafted the manuscript. JDL analyzed the data. EA conceived of the study, designed the experiment, coordinated and supervised the work and wrote the manuscript in its final form. All authors read and approved the final manuscript.
